# Silver nanoparticles and platelet-rich fibrin accelerate tendon healing in donkey

**DOI:** 10.1038/s41598-023-30543-w

**Published:** 2023-02-28

**Authors:** Mohammed A. H. Abdelhakiem, Ayman Hussein, Samia Moustafa Seleim, Abdelbaset Eweda Abdelbaset, Mahmoud Abd-Elkareem

**Affiliations:** 1 Department of Surgery, Anesthesiology, and Radiology, Faculty of Veterinary Medicine, Assiut University, Assiut, 71526 Egypt; 2 Clinical Laboratory Diagnosis, Department of Animal Medicine, Faculty of Veterinary Medicine, Assiut University, Assiut, 71526 Egypt; 3 Department of Cell and Tissues, Faculty of Veterinary Medicine, Assiut University, Assiut, 71526 Egypt

**Keywords:** Biotechnology, Cell biology, Medical research, Materials science

## Abstract

This study investigated the effect of the silver nanoparticles (AgNPs) and platelet-rich fibrin (PRF) in the healing of the severed superficial digital flexor tendon in donkeys (SDFT). Twenty-seven adult donkeys were used in the study. The animals were divided into three equal groups. The first group (control group) in which the severed SDFT was sutured without the addition of any adjuvant. In the second group, there was a suture of severed SDFT with the addition of 1 ml of 1 mM silver nanoparticles (AgNPs group). The third group was subjected to the cutting of SDFT and then the addition of PRF after its suture. Each group of animals was divided into three equal subgroups that were examined after 1, 2, and 3 months. Each group of animals was clinically evaluated by assessing lameness. Gross and microscopic examinations of the healed tendons were performed after 1, 2, and 3 months of surgery. In comparison to the control group, the lameness degree decreased in the PRF and AgNPs groups, particularly in the third month after surgery. Furthermore, the lameness decreased significantly after the 3rd month relative to the 1st-month lameness in the AgNPs group. Interestingly, it was found that the PRF and AgNPs enhanced cell alignment and collagen deposition at the site of tendon injury, particularly among third-month subgroups. Therefore, it could be concluded that the PRF and AgNPs are effective materials for enhancing SDFT healing in donkeys.

## Introduction

The superficial digital flexor tendon (SDFT) is one of the strongest tendons in the equine body. Its great tensile strength and low extensibility allow it to act as a force transmitter and dynamic amplifier during rapid muscular contractions^1^. Injuries of the superficial digital flexor tendon in the equine are numerous and common as a result of the nature of their work, such as pulling on hard carts, running ground, and repeated trauma from the toes of the hindlimbs^2,3^. The superficial digital flexor tendon of the forelimb at the mid-metacarpal region is most frequently affected. The lesion varies in severity from minor partial rupture to complete rupture of the tendon^4,5^.

The healing of tendons is time-consuming and is associated with high costs for treatment and rehabilitation of the animal^6,7^. Tendon injuries require a minimum of 3–6 months of restricted activity to allow sufficient time for healing^8^. It is generally accepted that the optimal treatment for complete flexor tendon lacerations includes debridement, suturing of the tendon ends, wound closure, and cast immobilization for at least 6 weeks^9,10^. Many trials have been performed over the years to treat tendon injuries, but there is little objective and conclusive evidence to support their clinical use^11^. Therefore, more effective novel strategies for tendon healing should be developed.

Silver nanoparticles (AgNPs) are very effective at promoting tissue healing. Besides exerting antimicrobial effects, AgNPs are also capable of accelerating burn wound healing, reducing wound inflammation, and modulating collagen deposition and alignment. Further, AgNPs stimulate the differentiation of fibroblasts in vitro^12–16^. Since fibroblasts are responsible for wound healing in the skin, meniscus, and tendons, AgNPs may aid in the healing of superficial flexor tendon injuries.

Platelet-rich fibrin (PRF) is a new generation of platelet concentrates with simplified processing and no biochemical blood handling^12^. It is a concentration of platelets on a fibrin membrane containing all the components of blood that are helpful for healing and immunity^13^. Although platelet and leukocyte cytokines play a role in PRF biology, the fibrin matrix represents the critical ingredient responsible for its real therapeutic potential^14^. PRF has concentrated platelet suspension in a small amount of plasma. Platelets contain important growth factors that increase cell proliferation, collagen production, chemotaxis, angiogenesis, and cell differentiation. Moreover, PRF contains three adhesive molecules (fibrin, fibronectin, and vitronectin) that have an important role in tissue healing^15^. PRF has been used to enhance the healing of bone^16,17^, and skin^18^ and is capable of increasing tendon cell proliferation over time^19,20^.

The purpose of this study was to investigate the role of the concentrated silver nanoparticles and platelet-rich fibrin in the healing of severed SDFT in donkeys, which can be used as a model for horse injuries.

## Materials and methods

### Animals

The experimental protocol was approved by the Local Ethical Committee and by the Institutional Review Board of the Faculty of Veterinary Medicine, Assiut University, and was carried out under relevant guidelines and regulations.

This work was done in compliance with the ARRIVE guidelines and regulations (https://arriveguidelines.org). All animals have been accommodated and cared for according to the Egyptian animal welfare law (No. 53, 1966).

A total of 27 clinically healthy adult donkeys were used in this study (13 males and 14 females). The animals ranged from 3 to 6 years old (mean = 4.4 ± 1.05) and weighed from 70 to 120 kg (mean = 99.5 ± 15.5). All animals were inspected clinically before the experiment to ensure that they were not lame. They were kept in the Veterinary Teaching Hospital (VTH), Faculty of Veterinary Medicine, Assiut University 2 weeks before the beginning of work. Food and water were introduced to animals ad libitum. The animals were randomly divided into 3 groups, each with 9 donkeys. Group 1: Full-thickness tenotomy of the superficial digital flexor tendon (SDFT) without treatment (control). Group 2: Full-thickness tenotomy of SDFT treated with silver nanoparticles (AgNPs). Group 3: Full-thickness tenotomy of SDFT treated with platelet-rich fibrin (PRF). Animals in each group were subdivided into 3 equal subgroups (n = 3). Subgroup 1: Animals were examined clinically and a tendon specimen was harvested after 1 month. Subgroup 2: Animals were examined and samples were obtained after 2 months. Subgroup 3: Animals were examined and tendon samples were harvested after 3 months.

### Pre-operative preparations

The physiological parameters including temperature, HR, RR, and capillary refilling time were evaluated in all animals. The posture and gait of animals were examined from all views. All the selected donkeys for this study had normal physiological parameters and were free from all external lesions especially those affecting the musculoskeletal system. The donkeys were dewormed using 40 g/100 K.B.W of piperazine citrate (Piperazine citrate 100%; UCCMA, Nasr City, Cairo, Egypt). The animals fasted for 8 h before the operation. The surgical site was prepared by clipping and shaving the hair, washing with soap and water, brushing with a soft brush, and then scrubbing with Povidone Iodine (Betadine^®^, El-nil Co., Egypt).

General anesthesia was performed using intravenous administration of 2 mg/K.B.W of xylazine Hcl (XYLA-JECT 2%, Adwia Pharmaceuticals Co. 10th of Ramadan city, Egypt) and 3 mg/kg.b.w of ketamine (Ketamine Rotexmedica 50 mg/ml, Arzneimittelwerk GmbH Rotexmedica, Germany). The animals were positioned in a lateral recumbence on the left side. Experimental operations were performed on the right forelimb in all groups of animals. A tourniquet was applied just below the carpal joint to control hemorrhage in the operated limb.

#### Preparation of silver nanoparticles (AgNPs)

**Figure 1:**
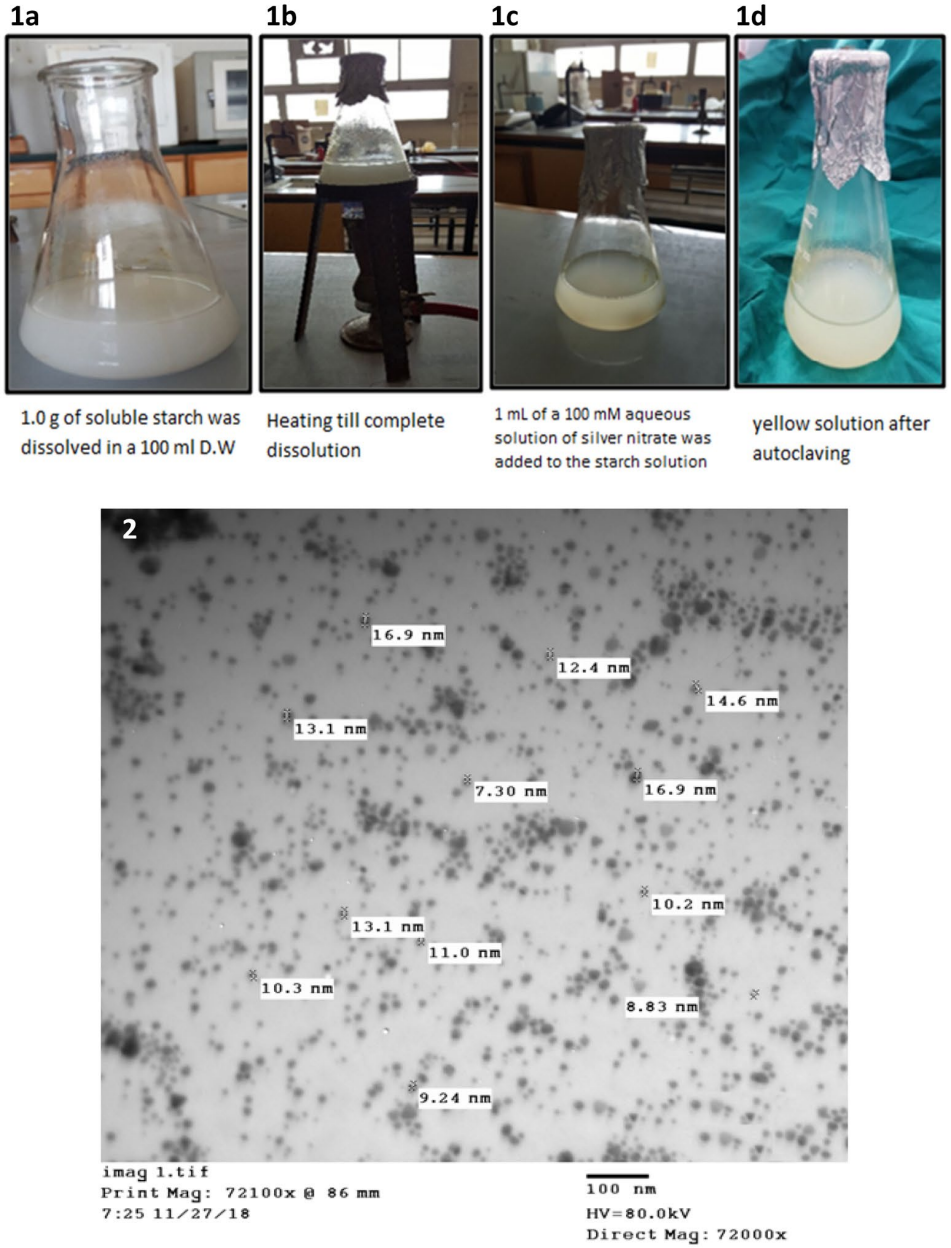
Preparation of the silver nanoparticles in the lab (**a–d**), and image of AgNPs under the electron microscope (TEM).

The solution was prepared according to Vigneshwaran et al.^21^. Briefly, in a 250 ml flask, 1.0 g of soluble starch (El-Gomhouria Co., Egypt) was added to 100 ml of distilled water and heated till complete dissolution. One ml of a 100 mM aqueous solution of silver nitrate (GAMMA Laboratory Chemicals., Egypt) was added to the starch solution and shacked well. This mixture was kept in an autoclave at 15 psi pressure, 121 °C for 5 min. The resulting solution was clear yellow indicating the formation of silver nanoparticles (Fig. 1).

The concentration of the resulting solution was estimated using a spectrophotometer (Graphite Furnace Atomic Absorption Model 210VGP) at the Chemistry Department, Faculty of Science, Assuit University. The concentration was 157 µM (157 ppm). The size of the silver nanoparticles was also estimated using transmission electron microscopy (TEM) (JEOL-JEM- 100CX II) at the Electron Microscopy Unit, Assuit University. The size was ~ 7.96 nm to 14.8 nm (figure).

#### Preparation of platelet-rich fibrin (PRF)

**Figure 2:**
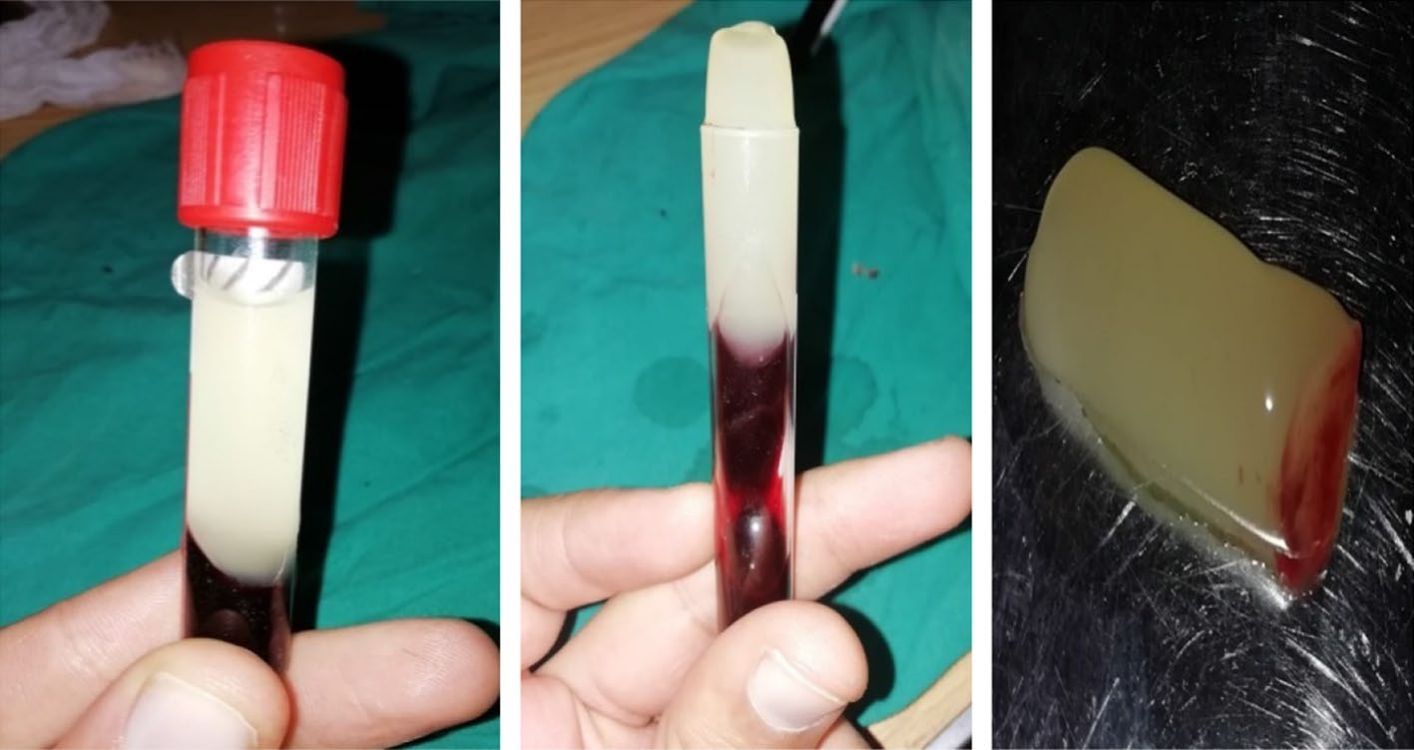
PRF formation after centrifugation of blood at 4000 rpm for 10 min. It looks like a jelly material.

It was prepared according to Choukroun et al.^22^ with a slight modifications. Briefly, ten ml of blood was collected from each donkey from the jugular vein and divided into two plain tubes (5 ml vacuum tubes, Biomedica Alex Co., Egypt) free from anticoagulants. They were immediately centrifuged (using a Hettich benchtop centrifuge D-78532, Germany) at a rate of 4000 rpm for 10 min. After centrifugation, the tube consists of three layers. The topmost layer represents acellular PPP (platelet-poor plasma). The PRF clot is in the middle while RBCs are present at the bottom of the test tube. The fibrin clot was collected from the tube and the attached red blood cells were scraped off and discarded (Fig. 2).

### Surgical technique

#### Group 1

Full-thickness tenotomy of SDFT without treatment (Control group).

SDFT was exposed through a linear skin incision at the lateral aspect of the mid-metacarpal region. The edges of the surgical wound were grasped using Allis forceps. The paratenon was dissected with scissors to identify the SDFT. Full-thickness tenotomy was performed using a scalpel. The two ends of SDFT were sutured with a double locking loop suture pattern according to Easley et al.^23^ using No. 2-0 nylon suture material (Ethicon/India). The paratenon was closed using a simple interrupted suture pattern and 3-0 polyglactin 910 (Egysorb; Taisier-Med). The subcutaneous tissue was sutured continuously using 3-0 Egysorb. The skin was closed in a simple interrupted pattern using braided silk no. 0 (Ethicon/India). The operated limb was kept in a cast using a combination of a splint and plaster of Paris bandage from the hoof to above the carpal joint for 1, 2, and 3 months in subgroups 1, 2, and 3 respectively.

#### Group 2

Full-thickness tenotomy of SDFT treated with AgNPs.

Full-thickness tenotomy of the superficial flexor tendon was performed, similar to group 1, but with the addition of AGNPS into the sutured tendon. A sterile insulin syringe and a hypodermic needle were used for the injection of silver nanoparticles. One ml of silver nanoparticles was injected under the paratenon after suturing (at and around the line of suturing). The concentration of Silver nanoparticles was 157 µM (157 ppm) and the size was ~ 7.96 nm to 14.8 nm.

#### Group 3

Full-thickness tenotomy of SDFT treated with PRF.

Full-thickness tenotomy of the superficial flexor tendon was performed as group 1. The PRF piece that was obtained after centrifugation of 10 ml of blood was used. The whole piece was put on the SDFT suture line of the tendon and was covered by paratenon and subcutaneous tissue.

### Post-operative care

The animals had received Penicillin-dihydrostreptomycin (Pen-Strep, Norbrook; 1 ml/25 kg I/M every day for 5 consecutive days) and Flunixin-meglumine (Flunixine; Norbrook; 1 ml/45 kg I/V daily for 3 successive days). The animals were kept in the stable. At 2 weeks postoperatively they were hand-walked for 10 min every day. Hand walking gradually increased in frequency and duration during the next weeks.

### Clinical evaluation

All animals were kept under complete observation after the operation. Animals were examined for lameness at 1, 2, and 3 months after the operation. Lameness was graded according to the American Association of Equine Practitioners (AAEP) on a scale of 0–5. Score 0: No lameness. Score 1: Lameness is difficult to observe and is not consistently apparent, regardless of circumstances (e.g. circling, inclines, hard surface, etc.). Score 2: Lameness is difficult to observe at a walk or when trotting in a straight line but consistently apparent under certain circumstances (e.g. weight-carrying, circling, inclines, hard surface, etc.). Score 3: Lameness is consistently observable at a trot under all circumstances. Score 4: Lameness is obvious at a walk. Score 5: Lameness produces minimal weight bearing in motion and/or at rest or a complete inability to move. The degree of lameness was determined at 1, 2, and 3 months in different subgroups.

### Gross evaluation

The healed SDFT was examined in its seat after the incision of the skin of anesthetized animals. The inclusion criteria for evaluation were tendon shape, size, adhesion, and color. A visual system score was used to evaluate the changes in the healed tendon. The symbols + , ++ , and +++ were used to refer to mild, moderate, and severe changes, respectively.

### Histopathological samples

The samples harvested for histopathological examination were: (1) Normal tendons from normal animals which had not been included in the study and had not been subjected to tenotomy (control negative). (2) Tendons were subjected to tenotomy and sutured without any adjuvant (control positive) at 1, 2, and 3 months post-operation. (3) Tendons were subjected to tenotomy and suturing with the addition of silver nanoparticles (AgNPs group) at 1, 2, and 3 months after surgery. (4) Tendons were subjected to tenotomy with the addition of Platelet-rich fibrin (PRF group) at 1, 2, and 3 months after the operation.

### Histopathological examination

The freshly excised tendon specimens were fixed in neutral buffered formalin 10% solution for 24 h at room temperature, dehydrated through ascending series of ethanol and cleared in methyl benzoate then embedded in paraffin wax. 5 μm paraffin sections were prepared and stained with the following histological stains: (1) Hematoxylin and Eosin for general histopathological examination^24^. (2) Crossmon’s trichrome technique for differentiation between mature and immature collagen fibers^25^. (3) Periodic acid Schiff (PAS) technique for demonstration of neutral mucopolysaccharides^26^.

### Statistical analysis

The obtained data were statistically analyzed using the statistical package for the Social Sciences for Windows (SPSS, version 21 (2016), Chicago, IL, USA). Univariate analysis of variances (ANOVA) with HSD–Tukey post hock multiple comparison test was used to determine the effect of time for each group and to determine the difference between groups at each time point. The lameness score data were analyzed in each group at different time intervals after surgery (1, 2, and 3 months) using one-way ANOVA to determine the effect of time on the lameness score and also the effect of treatment on the lameness score. One-way ANOVA was also used for the detection of the differences in the lameness score between different groups (treatments) at 1, 2, and 3 months. While two-way ANOVA was used to detect the effect of the time and treatment on the lameness score. The data were expressed as mean ± SE. Differences were considered significant when P < 0.05.

## Results

There is a lack of significant changes in the lameness score between donkeys at different times (one, two, and three months) in the control group. In the SNP group, there was a significant decrease in lameness after the 3rd month when compared to the 1st month (P value = 0.01). There were no significant differences in lameness score between donkeys at different times (1, 2, and 3 months) in the PRF group.

There were no significant changes in the lameness score between different groups at the 1st and 2nd months, but significant changes between the control and each of the AgNPs and PRF groups were detected after the 3rd month (P value = 0.05).

According to the results of two-way ANOVA, the time had a significant effect (P value = 0.008) on the lameness score. However, the treatment (P value = 0.052), and time treatment interaction (P value = 0.906) did not influence the lameness score.

### Gross evaluation of healed tendons

The gross evaluation of healed tendons in different groups after 1, 2, and 3 months post-surgery were recorded in Table 1.

**Table 1:** The gross changes in the healed SDFT in different groups at different times.

Group	Parameter	1 month	2 months	3 months
Control	Shape (distortion)	++	++	+
	Size (edematous)	+++	++	+
	Adhesion	++	++	+
	Color	Reddish	Reddish	opaque
AgNPs	Shape (distortion)	++	+	+
	Size (edematous)	++	+	+
	Adhesion	++	+	+
	Color	Less red	Less red	Less red
PRF	Shape (distortion)	+++	++	+
	Size (edematous)	+++	++	+
	Adhesion	+++	++	+
	Color	More red	Reddish	Less red

Visual inspection and evaluation was carried out. + (mild), ++ (moderate), and +++ (severe).

### Histopathological evaluation

**Figure 3:**
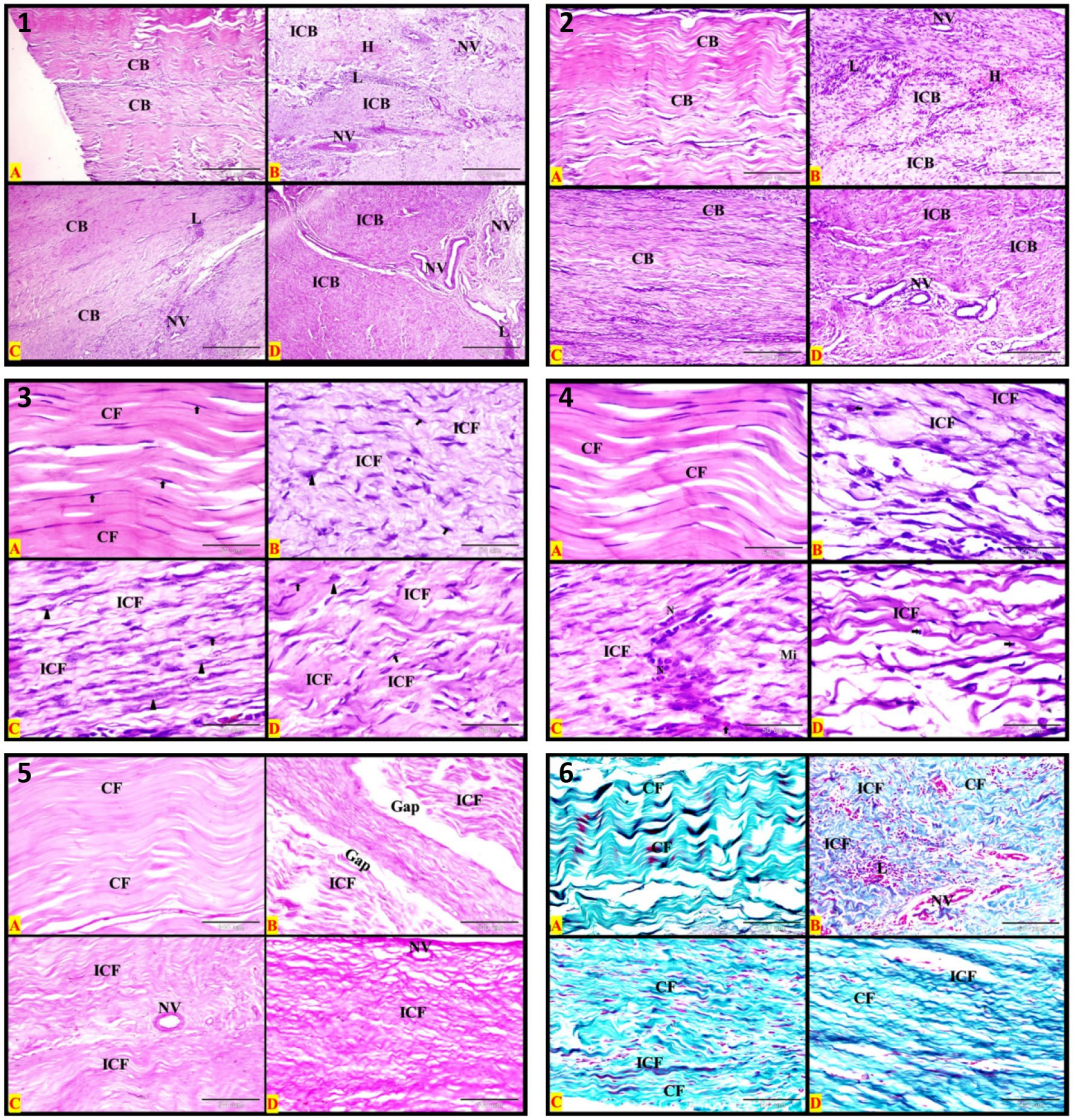
Photomicrograph of longitudinal section from the SDFT at 1 month post-injury. (**A**) Negative control, (**B**) positive control, (**C**) PRF treated, and (**D**) AgNPs treated. Plates (1–4): Haematoxylin and eosin stains, Plate (5): PAS & Haematoxylin, and Plate (6): Crossmon’s trichrome stain. *CB* regular collagen bundles, *ICB* irregular bundles, *H* hemorrhage, *L* inflammatory leucocytic infiltration, *NV* neovascularization (new blood vessels formation). All details of figures are displayed in Table 2. Plates 1, 2: Bars = 500 μm (40×); Plates 3, 4, 6: Bars = 50 μm (400×); Plate 5: Bars = 100 μm (200×).

**Figure 4:**
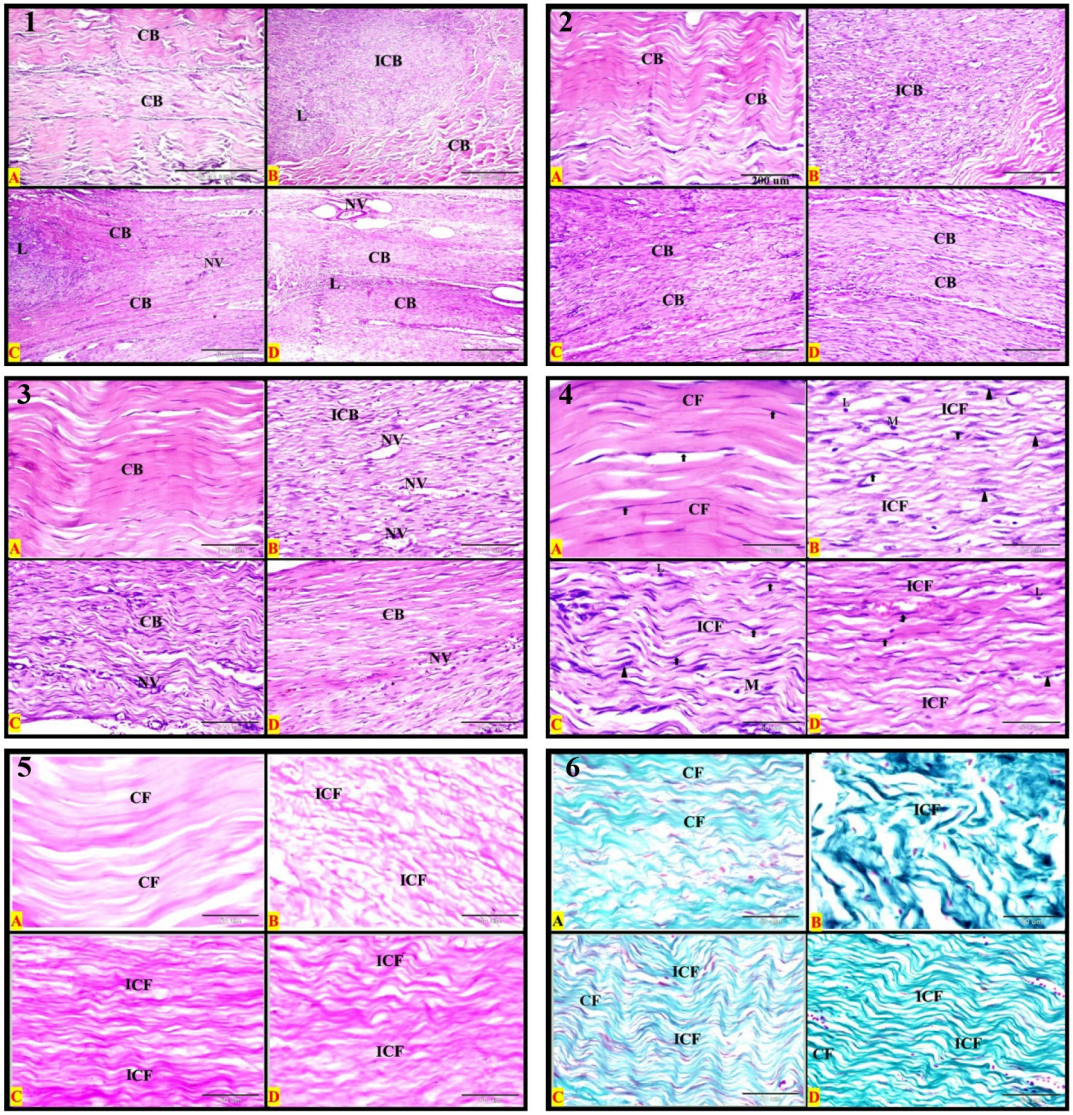
Photomicrograph of longitudinal section from the SDFT at 2 month post-injury. (**A**) Negative control, (**B**) positive control, (**C**) PRF treated, and (**D**) AgNPs treated. plates (1–4): Haematoxylin and eosin stains, Plate (5): PAS & Haematoxylin and plate (6): Crossmon’s trichrome stain. *CB* regular collagen bundles, *ICB* irregular bundles, *H* hemorrhage, *L* inflammatory leucocytic infiltration, *NV* neovascularization (new blood vessels formation). All details of figures are displayed in Table 2. Plates 1, 2: Bars = 500 μm (40×); Plates 3, 4, 6: Bars = 50 μm (400×); Plate 5: Bars = 100 μm (200×).

The changes of the healed SDFT in different groups at different times were recorded and shown in the histopathological figures using different stains. The normal tendon (control negative) showed parallel regularly arranged mature collagen bundles with no inflammatory cells. The tendon showed a definite pattern of the dense regular fibrous connective tissue arrangement. The parallel eosinophilic regularly arranged collagen bundles were separated by sparse matrix and regularly arranged rows of inactive fibrocytes; one row for each bundle. Each collagen bundle was formed of densely packed acidophilic parallel regularly arranged mature collagen fibers (Figs. 3, 4: A1, A2, A3, and A4). The control negative SDFT showed densely packed weak PAS-positive regular arranged mature collagen fibers (Figs. 3, 4: A5). By using Crossmon’s trichrome stain, we found that the control negative tendon had mainly densely packed regularly arranged mature collagen fibers with green color (Figs. 3, 4: A6).

**Figure 5:**
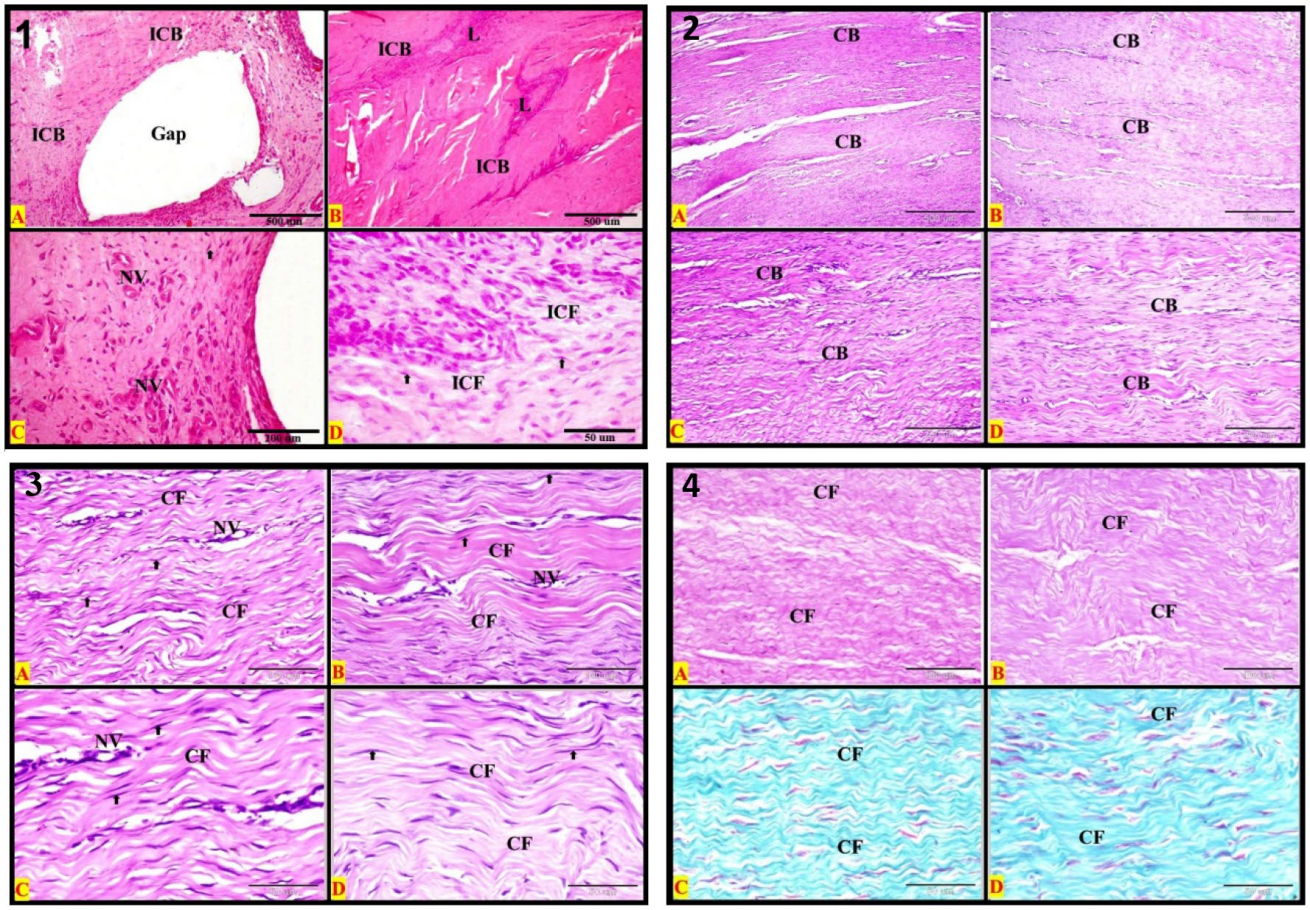
Photomicrograph of longitudinal section from the SDFT at 3 months post-injury. Plate (1): Positive control; Plates (2, 3 and 4); (**A,C**) PRF treated, and (**B,D**) AgNPs treated. Plates (1–3): H&E stains; Plate (4 **A,B**): PAS & Haematoxylin; Plate (4 **C,D**): Crossmon’s trichrome stain. *CB* regular collagen bundles, *ICB* irregular bundles, *H* hemorrhage, *L* inflammatory leucocytic infiltration and (NV): neovascularization (new blood vessels formation). All details of figures are displayed in Table 2. Plates 1, 2, 4: Bars = 500 μm (40×); plate 3: Bars = 100 μm (200×).

The findings of histopathological examination of the sutured tendons in different groups at different times were summarized in Table 2 and illustrated in Figs. 3, 4: B1–B6, Fig. 5 (A1–D1) (control positive) and Figs. 3, 4: C1–C6, Fig. 5: A2, 3, 4, C2, 3, 4 (PRF group) and Figs. 3, 4: D1–D6, Fig. 5: B2, 3, 4 & D2, 3, 4 (AgNPs group).

**Table 2:** The histopathological findings of the different groups at different times using three different stains.

Group	Month	H&E stain	PAS stain	Crossmon’s trichrome stain
Control	1st	Irregular arranged collagen bundles Each bundle has irregular fibers Hemorrhage	*Irregular* arranged strong *PAS-positive* immature collagen fibers	*Irregular* arranged *immature* collagen fibers
		Inflammatory L. infiltration Neovasularization The collagen bundle were not dense *More* inactive *fibrocytes* Few active fibroblasts Some *esinophiles* were detected at suturing site	Gabs of non-healing were observed between collagen bundles	
Control	2nd	As in the 1st month except *More* active *fibroblasts* Few inactive fibrocytes *Macrophages and lymphocytes* were detected at the suturing site	*Irregular* arranged strong *PAS-positive* collagen fibers	*Irregular* arranged *immature* collagen fibers
Control	3rd	Inactive fibrocytes were common Irregular arranged collagen fibers		
AgNPs	1st	Irregular arranged collagen bundles Inflammatory L. infiltration Neovasularization Sparsely packed bundles of collagen fibers *More* inactive *fibrocytes* Few active fibroblasts Some *lymphocytes* were detected at suturing site	*Irregular* arranged strong *PAS-positive* immature collagen fibers	There is a *mix* of immature and mature collagen fibers
AgNPs	2nd	As in the 1st month except *Regular* arranged collagen bundles	*Regular* arranged strong *PAS-positive* immature collagen fibers	There is a *mix* of immature and mature collagen fibers
AgNPs	3rd	Regular arranged collagen bundles Regular arranged mature collagen fibers within the bundle Inactive fibrocytes Densely packed bundles	Regular arranged *weak* PAS-positive immature collagen fibers	Mainly mature collagen fibers
PRF	1st	Somewhat regular arranged immature collagen fibers Inflammatory L. infiltration Neovasularization *More* active *fibroblasts* Few inactive fibrocytes Sparsely packed bundles of collagen fibers Neutrophiles, esinophiles and fibroblasts with mitotic figures were detected at the suturing site	*Somewhat regular* arranged *moderate PAS-positive* immature collagen fibers	There is a *mix* of immature and mature collagen fibers
PRF	2nd	As in the 1st month except More inactive fibrocytes Few active fibroblasts Macrophages and Lymphocytes were demonstrated at the site of suture	*Regular* arranged *strong PAS-positive* immature collagen fibers	There is a *mix* of immature and mature collagen fibers
PRF	3rd	Regular arranged collagen bundles Each bundle has regular arranged mature collagen fibers Inactive fibrocytes The bundles are densely packed	Regular arranged *weak* PAS-positive immature collagen fibers	Mainly mature collagen fibers within the tendon

## Discussion

The present study discussed the effect of silver nanoparticles and platelet-rich fibrin as promoting materials for healing of the superficial digital flexor tendon as a commonly injured tissue within the equine body. The donkeys were used in this study for the following reasons: First, it is a draft and working animal that is prone to tendon injury due to overuse. The second reason is that the donkey is considered a good model for horse injuries.

The results of this study revealed the promoting effect of both of AgNPs and PRF in the healing of the surgically severed SDFT in donkeys. Except for a few differences noted in the histopathological examination, both materials produced nearly identical results. As a result, the authors were unable to determine which was superior. However, PRF is commonly used by researchers. This is because PRFs were easier, faster, and less expensive to prepare than AgNPs.

Although clinical findings and gross appearance of injured tendons are very important in the detection of tendon changes, we relied on histopathology to investigate the effect of AgNPs and PRF in tendon healing. This could be attributed to a variety of injuries and changes of SDFT that occur silently without obvious clinical manifestations such as lameness and swelling or even on ultrasound^2,27,28^.

In the PRF group, there were no significant differences in the lameness scores at different intervals (1, 2, and 3 months). It may be explained by the PRF’s good effects on the severed tendon^29^, which were visible after the first month of treatment. This effect is completely supported by histopathological findings. The collagen bundles formed from regularly arranged collagen fibers in the tendon samples that were harvested after 1 month. These fibers after that had changed from immature to mature gradually with time which gained support and integrity for the tendons. The more interesting finding was that the most common cells found at the site of suturing were active fibroblasts, particularly in one-month tendon samples. These cells decreased gradually and were replaced by inactive fibrocytes. In the control group, the fibroblasts increased only in the two months samples. In the AgNPs group, there were few active fibroblasts at each time interval, though the deposition of regularly arranged mature and immature collagen appeared obviously in the 2- and 3-months tendon samples. Previous studies recorded the low activity of cells in SDFT which was subjected to damage^30,31^. They attributed this to two main hypotheses. Structures that require high mechanical strength to function, and high energy-storing tendons such as SDFTs, may be protected from remodeling because it may weaken them transiently due to the degradation and replacement of collagen molecules may occur. The second is that high-intensity exercise may reduce the tendon’s cell activity by altering the physicochemical or mechanical environment of these cells^31,32^.

The increased numbers of active fibroblasts, fibroblasts with a mitotic figure, and inflammatory cells at the point of tendon healing in the PRF group, especially after one month of injury, may have resulted from prolonged increases in concentrations of growth factors released from platelets. The most common is transforming growth factor (TGF)-β1 which plays an important role in the initial stages of wound healing through the mobilization of inflammatory cells and fibroblasts and the promotion of collagen production at late stages^33^.

A previous study found that PRF had no significant effect on the healing of flexor tendons in rabbits. PRF did not have a major influence on cellular organization. Furthermore, it had a negative impact on the biomechanical properties of the flexor tendons in rabbits^34^. The authors measured the healing effect of PRF 3 weeks, post-injury^34^. In the present study, the healing effect of PRF was examined 1, 2, and 3 months post-injury. Another study conducted on the patellar tendon in dogs revealed that the PRF membrane did not enhance the rate and quality of healing of the central defect in the patellar tendon at 4 and 8 weeks after surgery^35^. On the other hand, several studies have shown that different growth factors and PRF enhance the healing of injured or severed tendons and other tissue^16,17,36–38^. As with PRP, PRF contains an abundance of growth factors and cytokines which are produced by platelets^39^. Some previous studies were conducted to investigate the effect of PRF on the healing of flexor and Achilles tendons in rabbit and rat models^20,40^. These studies have revealed that healing of the tendon was better in the first 2 weeks, and there were no significant changes in the weeks after. In the present study, there were substantial changes in the healing of the tendon treated by PRF, especially at the third month. Adhesion, distortion, and edema were recorded in the PRF-treated tendons which gradually subsided with time. The distortion, edema, and adhesion were severe at 1 month and mild at 3 months. These undesirable effects of PRF in the first 3 weeks of tendon healing were recorded in a previous study. All adhesion, edema, inflammation, and tendon bulkiness hinder the tendon gliding and eventually the tendon healing^34^. The authors attributed these tendon changes to the applied PRF. As PRF is an autologous substance, it caused severe tissue reactions in the treated tendon and surrounding tissue that included inflammation, adhesions, edema, and distortions.

According to previous studies^41–44^, collagen deposition and angiogenesis are considered important factors for tendon healing. Another study^29^ found that PRF increases the production of different types of tendon proteins, especially collagen. The collagen and protein synthesis may be attributed to the growth factors derived from the platelets in PRF, especially the platelet-derived growth factor (PDGF). Furthermore, the abundant cells of fibroblasts are responsible for collagen synthesis^41,45^. In the cell culture experiment, the use of biodegradable fibrin with PRF exudate increased the components of the extracellular matrix (glycosaminoglycan, glycoprotein) and collagen type II when the chondrocytes were cultured^46^. In a previous study in a rabbit model, the PRF promoted the formation of type I and II collagen in the osteochondral defects^47^.

The results of the present study revealed the deposition of collagen was gradually increasing with time, especially the mature type in both of PRF and AgNPs groups. Additionally, the orientation and arrangement of collagen fibers were regular which gradually increased the density of collagen bundles. The inflammatory cells and neovascularization were high in the first 2 months and then decreased in the third month. The fibroblasts were abundant relative to the fibrocytes for collagen deposition in the first 2 months, and then in the third month, the latter cells were abundant. All of the aforementioned results refer to the good accelerating effect of PRF and AgNPs on tendon healing. Additionally, the remodeling of the collagen fibers began from the first month, either due to the deposition of immature and mature fibers or because of regularly arranged fibers. These findings of the healed tendons were mostly consistent with the findings which have been recorded in the previous studies^34,48,49^.

The present study revealed that the silver nanoparticles (AgNPs) improved the healing of the sutured SDFTs in donkeys. Their effects were close to the effect of PRF. Clinically, there were no significant differences in the lameness score between the PRF and AgNPs. But a significant decrease in the lameness score compared to the control group in the third month was recorded in AgNPs and PRF groups. Furthermore, the lameness was substantially diminished in AgNPs group after the third-month examination relative to the first-month examination. Although gross changes of the healed tendon were observed at 1 month examination in the PRF group, there was no significant difference in the lameness score between the 1st and 3rd month examinations. This refers to the good and accelerating healing effect of PRF relative to AgNPs. These results were confirmed on the microscopic examination. The edema, distortion, adhesion, and tendon bulkiness were mild in the tendons that were treated with AgNPs than in PRF-treated tendons. This is due to the nature of the two added materials. The PRF is considered a biological material, which may be associated with severe tissue reaction^34^, while the AgNPs are synthetic chemical materials, which reacted low with the viable tissue. Moreover, the AgNPs has an anti-inflammatory effect^48,49^. It is well known that the healing process in tendons is accompanied by inflammation. The macrophages come to the site of inflammation from the blood and released a pro-inflammatory cytokine called tumor necrosis factor-alpha (TNF-α)^50^. The latter stimulates the release of other cytokines and metalloproteinases^51^. They collectively lead to the degradation of the tendon extracellular matrix. The AgNPs suppress the effect of the macrophages at the seat of tendon injury, which in turn leads to the stop of releases of pro-inflammatory cytokines and metalloproteinases. Therefore, the adverse effects of the macrophages and their products on the tendon will be stopped eventually^48,49^. According to these aforementioned results, it is believed that the anti-inflammatory property, antibacterial effect, and the stimulation of cell proliferation are the main mechanisms of the action of silver nanoparticles that leads to an acceleration in healing, better collagen and proteoglycan deposition, and increased strength in the healed tendon^49^. In the present study, the histopathological examination of the healed tendons treated with AgNPs showed a gradual increase in collagen deposition from the 1st to the third month. In addition, the inflammatory cells were abundant in the 1st-month treatment subgroup and then gradually subsided to be low or absent in the third month of treatment. These histopathological findings demonstrated and verified the anti-inflammatory property of AgNPs, as well as their ability to accelerate tendon healing. Our results of AgNPs were consistent with the findings of other studies^48–50,52^ that evaluated the effect of AgNPs on the healing of different tissues including tendons.

### Study limitations

The lack of measurement of the tensile strength of the healed tendon in all groups was a limitation in our study, so a further study should be conducted on the tendons with consideration of the measurement of tensile strength using biomechanics. In addition, the positive control samples of the 3rd month were not stained using PAS and Crossman’s Trichome due to the spoilage of samples. Only the H&E-stained samples had been examined. After obtaining histological findings, the authors thought that the study should be extended longer than 3 months (6 months at least). Therefore, a further study is recommended to measure the tensile strength and inspect the microscopic changes that will occur in the severed SDFT treated with AgNPs and PRF for 6 months. Furthermore, we examined the in vivo effects of AgNPs and PRF on tendon healing. However, in vitro experiments were not simultaneously conducted.

## Conclusion

The current findings suggest that the effects of PRF and AgNPs on tendon healing in donkeys are nearly identical. Both materials were used once and produced satisfactory results in the healing of the superficial flexor tendon in donkeys. The PRF is easy to prepare but it is associated with severe inflammatory reactions, especially in the first few weeks of its application. Although the AgNPs are time-consuming to prepare, somewhat expensive and require a special procedure for preparation and characterization (appropriate size and concentration), they have potent anti-inflammatory and anti-bacterial effects. NSAIDs should be given to PRF-treated animals, especially during the initial treatment period (first 2 weeks).

## Data Availability

The datasets used and/or analysed during the current study available from the corresponding author on reasonable request.
